# Handshaking for ultrafast endocytosis: Dynamin1xA and Endophilin A1 sealed the deal

**DOI:** 10.1038/s44318-024-00179-1

**Published:** 2024-07-23

**Authors:** Santiago López-Begines, Rafael Fernández-Chacón

**Affiliations:** 1 Instituto de Biomedicina de Sevilla (IBiS, Hospital Universitario Virgen del Rocío/CSIC/Universidad de Sevilla), Dpto. de Fisiología Médica y Biofísica, Facultad de Medicina, and CIBERNED ISCIII, Seville, Spain; 2 Luxembourg Centre for Systems Biomedicine, University of Luxembourg, Belvaux, Luxembourg

**Keywords:** Membranes & Trafficking, Neuroscience, Organelles

## Abstract

A new study reveals how preassembled components at the endocytic zone permit ultrafast endocytosis to occur so quickly.

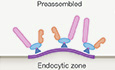

## Teaser

Brain function relies on quick inter-neuron communication at specialized points of contact termed synapses. In the latest issue of The EMBO Journal, Imoto, Xue, et al (2024) report the discovery of a novel, regulated interaction between two major endocytosis players which supports the notion of a preassembled protein machinery at presynaptic nerve terminals that can explain how the high speed of ultrafast endocytosis is possible.

From basic reflexes to complex cognitive processes, the central nervous system’s operation relies on neuronal communication mediated by fast synaptic transmission. At the nerve terminals, synaptic vesicles loaded with neurotransmitters are docked at the so-called active zone of the presynaptic membrane. Upon the arrival of an action potential (a nerve impulse), calcium influx through specific ion channels triggers neurotransmitter release in less than a millisecond through synaptic vesicle exocytosis (Südhof, 2013). The preassembly of the sophisticated active-zone protein machinery, which connects synaptic vesicles to calcium channels, is essential for fast release through the fusion pore (Emperador-Melero and Kaeser, 2020). Exocytosis must be coupled to endocytosis to retrieve and recycle plasma membrane and to refill synaptic vesicles essential to sustain neurotransmitter release. Several modes of synaptic endocytosis have been described, including the closing of the fusion pore (kiss-and-run), clathrin-mediated endocytosis, activity-dependent bulk endocytosis, and more recently, ultrafast endocytosis (Chanaday et al, 2019).

Ultrafast endocytosis was originally described in cultured mouse neurons obtained from the hippocampus, a well-structured region of the brain with a major role in learning and memory (Watanabe et al, 2013, 2014). The discovery of ultrafast endocytosis was attained through a sophisticated combination of neuronal stimulation with optogenetics (light-induced opening of light-sensitive ion channels), coupled with rapid high-pressure freezing fixation ideal for electron microscopy (EM) analysis. With this ‘flash and freeze’ approach, it was possible to capture EM images of synaptic endocytosis at specific time points after stimulation. Watanabe and colleagues described endocytic pits and plasma membrane fission events occurring as soon as 50 ms following a single action potential. Strikingly, those events turned out to be independent of clathrin, the typical molecular scaffold mediating vesicular uptake of cargo at the plasma membrane (Watanabe et al, 2013, 2014). In contrast, the mechanochemical enzyme Dynamin1 (Dyn1) (Ferguson and De Camilli, 2012), which mediates vesicular fission, and other endocytic proteins were required for ultrafast endocytosis (Watanabe et al, 2018). Interestingly, there are four Dyn1 variants (xA, xB, xC, and xD) generated by alternative splicing of its C-terminus. The variants Dyn1xA and Dyn1xB mediate synaptic vesicle endocytosis, but only Dyn1xA is required for ultrafast endocytosis (Imoto et al, 2022). For ultrafast endocytosis to occur, the endocytic machinery must be ready on the plasma membrane immediately after synaptic vesicle fusion. In a recent study, Watanabe’s group provided strong evidence that the endocytic machinery is preassembled by demonstrating that Dyn1xA is pre-recruited to endocytic sites for ultrafast endocytosis. Their evidence suggests that Dyn1xA is recruited through the interaction with the endocytic protein Syndapin 1 to form molecular condensates on the plasma membrane (Imoto et al, 2022), however, the variant Dyn1xB, that also binds Syndapin 1, was not localized to endocytic zones. Therefore, the molecular mechanism underlying the pre-deployment of Dyn1xA, mandatory to trigger ultrafast endocytosis, has remained so far unsolved. Now, in an elegant and thorough study published in The EMBO Journal (Imoto, Xue et al, 2024), the groups of Phillip J. Robinson and Shigeki Watanabe demonstrate an unexpected and specific interaction of Dyn1xA, but not Dyn1xB, with the endocytic protein Endophilin A, providing a robust and convincing molecular framework to understand the operation and regulation of ultrafast endocytosis.

**Figure 1:**
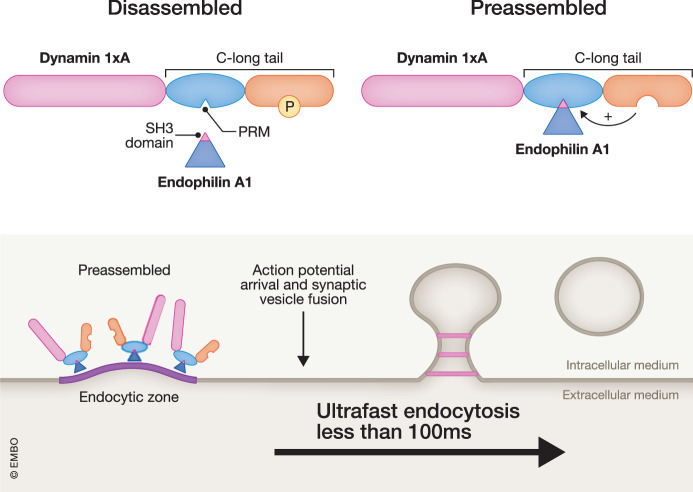
Preassembly of Dynamin1xA and Endophilin A1 at the endocytic zone mediates ultrafast endocytosis. Specific amino acids at the C-terminal long tail (C-long tail) of Dyn1xA promote the high-affinity interaction between the Endophilin A1 SH3-domain and a proline-rich motif (PRM) also located at the C-terminal long tail of Dyn1xA. The dephosphorylation of the Dyn1xA long tail enhances the interaction with Endophilin A1 and promotes the preassembly at the endocytic zone that becomes ready to go upon synaptic vesicle fusion speeding ultrafast endocytosis. Proteins length and domains are not to actual scale.

Dyn1 contains an N-terminal GTPase domain and a C-terminal proline-rich domain (PRD). The PRD is formed by multiple proline-rich motifs (PRM) that interact with the C-terminal Src-homology 3 (SH3) domains of key endocytic proteins such as Amphiphysin 1, Syndapin 1, and Endophilin A. These three proteins sense membrane curvature and tubulate membranes through their N-terminal Bin/Amphiphysin/RVS (BAR) domain. In addition, at endocytic sites they form a molecular scaffold to recruit Dyn1 and execute membrane fission through GTPase activity. Dyn1xA and Dyn1xB differences occur at the C-terminus after the residue P844: Dyn1xA contains a long tail extension of 20 unique amino acids, while Dyn1xB contains a shorter tail of only 7 unique amino acids. Furthermore, the long tail extension includes three additional PRMs which could expand the repertoire of interactions of Dyn1xA with SH3-domain containing proteins (Fig. 1). The binding of both Dyn1 variants to endocytic proteins is activated by calcineurin-mediated dephosphorylation induced by neuronal activity. Calcineurin dephosphorylates the residues located at specific Dyn1 regions called phosphoboxes: phosphobox-1 (residues S774 and S778) and phosphobox-2 (residues S851 and S857 only present in Dyn1XA) (Fig. 1). Intriguingly, the function of phosphobox-2 was, up to now, unclear but Imoto, Xue et al have now found a key role in the regulation of ultrafast endocytosis. There are remarkable differences between Dyn1xA and Dyn1xB. For example, Dyn1xA localizes to endocytic sites and is essential for ultrafast endocytosis, while Dyn1xB is predominantly cytoplasmic and does not participate in ultrafast endocytosis but rather in activity-dependent bulk endocytosis. Reasoning that the functional differences between Dyn1xA and Dyn1xB could be explained by variant-specific interactors, Imoto, Xue et al used recombinant proteins encoding the complete PRDs of Dyn1xA and Dyn1xB to affinity purify specific bindings partners from rat nerve terminal (synaptosomes) lysates. This approach led to the discovery of a novel and specific high-affinity (nanomolar) binding site for Endophilin A1 at the Dyn1xA long tail (Fig. 1). This site is different to a previously known Endophilin A1 binding site common to both Dyn1xA and xB. Upon a systematic study based on pull downs of recombinant proteins, site-directed mutagenesis, mass spectrometry analysis, nuclear magnetic resonance (NMR), and chemical shift perturbation (CSP) the authors remarkably found that Dyn1xA’s long tail contained a specific SH3-binding PRM, absent in Dyn1xB and flanked by specific amino acids that were critical to enhance Endophilin A binding. Interestingly, the dephosphorylation of some of those amino acids (S851/857), located downstream at phosphobox-2, increased Endophilin A1 binding (Fig. 1) while phospho-mimetic mutations (S851/857D) blocked its binding. Further, the authors remarked that the Down Syndrome and autism-related kinase DYRK1 phosphorylates the Dyn1xA at S851 (Huang et al, 2004), suggesting that DYRK1-dependent alterations and consequences on ultrafast endocytosis in brain disorders deserve investigation. Alanine substitution of one of those residues (R846A) upstream of the PRM blocked Endophilin A1 binding.

In previous studies, the authors suggested that Dyn1xA forms condensates that are enriched at endocytic zones (Fig. 1), just outside the active zone (Imoto et al, 2022). To investigate if the Dyn1xA long tail was associated with Endophilin A1 accumulation at endocytic zones, they overexpressed versions of Dyn1xA and Endophilin A1 (and A2) fused to fluorescent reporters (GFP-Dyn1XA and Endophilin A1-mcherry or Endophilin A2-mcherry), visualized synaptic boutons with superresolution microscopy and quantified the distance between the Dyn1xA and Endophilin A puncta. They found that more that 70% of Dyn1xA puncta contained Endophilin A and Dyn1xA puncta localized to a putative ideal site for ultrafast endocytosis: the edge of the active zone. Interestingly, Dyn1xA mutants defective for Endophilin A binding (Dyn1XA-S851/857D and Dyn1XA-R846A) were not near the active zone but were broadly distributed elsewhere. These measurements are the first evidence indicating that the protein-protein interactions found in vitro between Dyn1xA and Endophilin A also occurred within the synaptic bouton.

The authors use their previously developed flash-and-freeze method (Watanabe et al, 2013, 2014, 2018; Imoto et al, 2022) to characterize the functional role of Dyn1xA, and Dyn1xA-mutant versions, in in vitro hippocampal neurons from mice lacking Dyn1 (*Dyn1* KO). As previously described (Watanabe et al, 2013; Imoto et al, 2022), endocytosis occurred in wild-type neurons as expected with ultrafast endocytosis occurring within the first 100 ms, followed by the endosomal uptake of endocytic vesicles (measured as the uptake of extracellular ferritin) at 1 s, and the formation of new synaptic vesicles at 10 s. In contrast, none of those events were detected in DynKO neurons, and only shallow and non-constricted endocytic pits were observed, indicating a total failure of endocytosis. Remarkably, the overexpression of Dyn1xA resulted in a near-complete rescue of ultrafast endocytosis. However, a defect in the formation of ferritin-positive endosomes persisted, which could be explained if other splice variants of Dyn1 participate in endosomal formation or the overexpression of Dyn1xA itself induces morphological alterations of endosomes. In any case, the overexpression of Dyn1xA-S851/857D or Dyn1xA-R846A did not rescue the phenotype, providing strong support that the interaction between Dyn1xA and Endophilin A is required for ultrafast endocytosis.

To further asses the role of Dyn1xA in the endocytosis dynamics of synaptic vesicle proteins, Imoto, Xue et al used imaging to monitor the exo- and endocytosis of the vesicular glutamate transporter 1 (vGlut1) in hippocampal cultures expressing vGlut1- pHluorin a version of vGlut1 fused to pHluorin (Voglmaier et al, 2006), a modified GFP that it is especially sensitive to pH. At the synaptic vesicles, the pHluorin moiety of vGlut1- pHluorin faces the vesicular lumen and changes its fluorescence during the synaptic vesicle cycle. In resting conditions, the vesicular intraluminal milieu is very acidic (pH ≈5.5) and quenches pHluorin. Upon exocytosis, protons escape to the extracellular space increasing pHluorin fluorescence and, after endocytosis, fluorescence decreases due to vesicle re-acidification. In Dyn1 KO cultures, neuronal stimulation with trains or single action potential resulted in slower and less frequent endocytosis, and was fully rescued in neurons overexpressing wild-type Dyn1xA. Consistently with the proposed mechanism for Dyn1xA in ultrafast endocytosis, Dyn1 KO neurons overexpressing Dyn1xA-S851/857D or Dyn1xA-R846A did not rescue endocytosis.

In summary, the regulated and high-affinity interaction between Dyn1xA and Endophilin A1 demonstrated by Imoto, Xue et al supports a model in which major players of the endocytic machinery are already pre-deployed and ready-to-go at endocytic zones without the need of time-consuming recruitment of proteins upon exocytosis (Fig. 1). Such a molecular mechanism would explain how ultrafast endocytosis can occur with a millisecond-scale delay after synaptic vesicle fusion, reinforcing the notion that ultrafast endocytosis is a key and major mechanism for synaptic vesicle recycling.

## References

[CR1] Chanaday NL, Cousin MA, Milosevic I, Watanabe S, Morgan JR (2019) The synaptic vesicle cycle revisited: new insights into the modes and mechanisms. J Neurosci 39:8209–8216 10.1523/JNEUROSCI.1158-19.2019 PMC6794917 31619489

[CR2] Emperador-Melero J, Kaeser PS (2020) Assembly of the presynaptic active zone. Curr Opin Neurobiol 63:95–103. 10.1016/j.conb.2020.03.008 32403081 10.1016/j.conb.2020.03.008 PMC7483790

[CR3] Ferguson SM, De Camilli P (2012) Dynamin, a membrane-remodelling GTPase. Nat Rev Mol Cell Biol 13:75–88. 10.1038/nrm3266 22233676 10.1038/nrm3266 PMC3519936

[CR4] Huang Y, Chen-Hwang M-C, Dolios G, Murakami N, Padovan JC, Wang R, Hwang Y-W (2004) Mnb/Dyrk1A phosphorylation regulates the interaction of Dynamin 1 with SH3 domain-containing proteins. Biochemistry 43:10173–10185 10.1021/bi036060+ 15287745

[CR5] Imoto Y, Raychaudhuri S, Ma Y, Fenske P, Sandoval E, Itoh K, Blumrich EM, Matsubayashi HT, Mamer L, Zarebidaki F et al (2022) Dynamin is primed at endocytic sites for ultrafast endocytosis. Neuron 110:2815–2835 35809574 10.1016/j.neuron.2022.06.010 PMC9464723

[CR11] Imoto Y, Xue J, Luo L, Raychaudhuri S, Itoh K, Ma Y, Craft GE, Kwan AH, Ogunmowo TH, Ho A, Mackay JP, Ha T, Watanabe S, Robinson PJ (2024) Dynamin 1xA interacts with Endophilin A1 via its spliced long C-terminus for ultrafast endocytosis. EMBO J. 10.1038/s44318-024-00145-x. Online ahead of print 10.1038/s44318-024-00145-x PMC11329700 38907032

[CR6] Südhof TC (2013) Neurotransmitter release: the last millisecond in the life of a synaptic vesicle. Neuron 80:675–690. 10.1016/j.neuron.2013.10.022 24183019 10.1016/j.neuron.2013.10.022 PMC3866025

[CR7] Voglmaier SM, Kam K, Yang H, Fortin DL, Hua Z, Nicoll RA, Edwards RH (2006) Distinct endocytic pathways control the rate and extent of synaptic vesicle protein recycling. Neuron 51:71–84 16815333 10.1016/j.neuron.2006.05.027

[CR8] Watanabe S, Mamer LE, Raychaudhuri S, Luvsanjav D, Eisen J, Trimbuch T, Söhl-Kielczynski B, Fenske P, Milosevic I, Rosenmund C et al (2018) Synaptojanin and endophilin mediate neck formation during ultrafast endocytosis. Neuron 98:1184–1197.e6 29953872 10.1016/j.neuron.2018.06.005 PMC6086574

[CR9] Watanabe S, Rost BR, Camacho-Pérez M, Davis MW, Söhl-Kielczynski B, Rosenmund C, Jorgensen EM (2013) Ultrafast endocytosis at mouse hippocampal synapses. Nature 504:242–247 24305055 10.1038/nature12809 PMC3957339

[CR10] Watanabe S, Trimbuch T, Camacho-Pérez M, Rost BR, Brokowski B, Söhl-Kielczynski B, Felies A, Davis MW, Rosenmund C, Jorgensen EM (2014) Clathrin regenerates synaptic vesicles from endosomes. Nature 515:228–233 25296249 10.1038/nature13846 PMC4291189

